# COVID‐19 patients in intensive care develop predominantly oliguric acute kidney injury

**DOI:** 10.1111/aas.13746

**Published:** 2020-11-28

**Authors:** Tomas Luther, Sara Bülow‐Anderberg, Anders Larsson, Sten Rubertsson, Miklos Lipcsey, Robert Frithiof, Michael Hultström

**Affiliations:** ^1^ Department of Surgical Sciences, Anaesthesiology and Intensive Care Medicine Uppsala University Uppsala Sweden; ^2^ Department of Medical Sciences Clinical Chemistry Uppsala University Uppsala Sweden; ^3^ Hedenstierna laboratory CIRRUS, Anaesthesiology and Intensive Care Medicine Department of Surgical Sciences Uppsala University Uppsala Sweden; ^4^ Department of Medical Cell Biology Integrative Physiology Uppsala University Uppsala Sweden

**Keywords:** acute kidney injury, biomarkers, COVID‐19, intensive care

## Abstract

**Background:**

Acute kidney injury (AKI) is a syndrome of reduced glomerular filtration rate and/or reduced urine flow associated with mortality in corona virus disease 2019 (COVID‐19). AKI is often associated with renal tissue damage, which may lead to chronic kidney disease. Biomarkers of tissue damage may identify patients of particular risk.

**Methods:**

In a prospective observational study of 57 patients admitted to intensive care, AKI incidence and characteristics was evaluated according to KDIGO criteria and related to days after admission. Urinary albumin, Neutrophil Gelatinase‐Associated Lipocalin (NGAL), Kidney Injury Molecule 1 (KIM‐1) and Plasma Tissue Inhibitor of MetalloProteinase 2 (TIMP‐2) were analysed in 52 patients at admission.

The majority (n = 51, 89%) of patients developed AKI, and 27 (47%) patients had predominantly oliguric AKI where oliguria was more severe than plasma Creatinine increase. Severe oliguria within first 2 days after admission was common (n = 37, 65%), whereas stage 2 and 3 AKI due to Creatinine occurred later than day 2 in 67% (12/18) of cases. Renal replacement therapy was started in 9 (16%) patients, and 30‐day mortality was 28%. Urinary biomarkers were increased in a majority of patients, but did not robustly predict KDIGO stage. Most patients had microalbuminuria, and severe albuminuria (albumin Creatinine ratio > 30 mg/mmol) was found in n = 9 (17%) patients.

**Conclusions:**

A majority of patients with COVID‐19 admitted to the ICU develop AKI. The functional deficit is often low urinary volume, and initial levels of biomarkers are generally increased without clear relation to final AKI stage.

**Ediorial Comment**

This study among critically ill COVID‐19 patients found that almost 90% in this cohort developed acute kidney. Several urinary biomarkers of kidney injury were found to be increased already at ICU admission and microalbuminuria was very common.

## 1 INTRODUCTION

The pandemic corona virus disease 2019 (COVID‐19) is caused by the severe acute respiratory syndrome coronavirus 2 (SARS‐CoV‐2).^1^ In regions particularly affected by SARS‐CoV‐2, intensive care unit (ICU) admission due to Covid‐19 has been dramatic, with a high proportion of patients requiring mechanical ventilation, long lengths of stay and with substantial mortality.^2^ Acute kidney injury (AKI) has been found to be an independent risk factor for death in hospitalized COVID‐19 patients.^3^ Early reports suggested that AKI only affects a small proportion of patients with COVID‐19, in one early report only 2.9% of patients with severe disease were affected.^4^ However, in New York 130 of the first 393 consecutive patients admitted to two hospitals required mechanical ventilation and 13.3% of these required renal replacement therapy (RRT) compared to 0.4% of non‐mechanically ventilated.^5^ Recent data show that in patients requiring mechanical ventilation up to 90% develop AKI.^6^, ^7^

There are several possible ways COVID‐19could cause AKI. The virus spike (S) glycoprotein binds to and uses human angiotensin‐converting enzyme 2 (ACE2) to enter the cells,^8^ a protein that is known to be expressed in the kidney.^9^ In addition, the consequences of acute respiratory distress syndrome (ARDS) development and treatment include release of cytokines, potential hypoperfusion, and fluid imbalance that have all been implicated in the development of AKI.^10^ Some patients develop severe hypernatremia associated with high renin and AKI in a way that may suggest extracellular fluid volume contraction.^11^ There is limited knowledge of the characteristics of AKI in COVID‐19 and whether renal injury biomarkers are useful for identifying patients with particular risk, and reflecting the pathophysiological mechanisms involved.

The aim of this study was to investigate the incidence and characteristics of AKI in COVID‐19 patients admitted to intensive care by comparing the KDIGO criteria for Creatinine increase and urine volume over time, as well as their relation to biomarkers for kidney tissue injury, glomerular and tubular function measured at admission.

## 2 MATERIALS AND METHODS

The study was approved by the National Ethical Review Agency (EPM; No. 2020‐01623). Informed consent was obtained from the patient, or next of kin if the patient was unable give consent. The Declaration of Helsinki and its subsequent revisions were followed. This is a sub‐study of an ongoing prospective observational study performed at the general ICU at the Uppsala University Hospital at the time treating primarily COVID‐19 patients. The protocol of the study was registered (ClinicalTrials ID: NCT04316884).

### 2.1 Data collection and patient groups

All adult patients with confirmed or suspected COVID‐19 admitted to the ICU were screened for inclusion. Exclusion criteria were pregnancy, breast‐feeding, pre‐existing end‐stage renal failure or dialysis. Data and medical history were collected from the electronic medical record. Plasma Creatinine (P‐Creatinine) were determined daily. Urine samples from the day of admission were used to analyze Creatinine, albumin, Cystatin C, Neutrophil Gelatinase‐Associated Lipocalin (NGAL), Kidney Injury Molecule 1 (KIM‐1) and Plasma Tissue Inhibitor of MetalloProteinase 2 (TIMP‐2). ICU days where standardized as a 24‐hour period between 7 am and 7 am with admission date assigned as ICU day 0. This to align in time observed clinical parameters with daily tests, which have been assigned to the start of the ICU day. Simplified acute physiology score 3 (SAPS 3) was used to grade the severity of disease and pre‐existing conditions. Fluid balance is defined as all fluids given either intravenously or orally with quantified losses from catheters and drains including urine output and fecal losses subtracted. No estimation to insensible losses were included.

### 2.2 Acute kidney injury definition and calculation

Acute kidney injury was calculated according to the KDIGO definition during ICU stay and the preceding hospitalization. Data were available up to 14 days after ICU admission at the time of analysis. Baseline P‐Creatinine was determined from the laboratory database as lowest P‐Creatinine value within 7 days of hospital admission, or, when lacking, in the year before hospitalization if available. Hourly urine output was recorded during ICU stay. Any period fulfilling the KDIGO urine output criteria was registered with the time to each stage of AKI. Time periods without complete registration were disregarded.

### 2.3 Laboratory analysis

P‐Creatinine was analyzed on an Architect ci16200 (Abbott Laboratories). Creatinine and Urea in urine were measured on a Mindray™ BS‐380 (Mindray Medical International) with reagents from Abbott Laboratories. Urine Cystatin C was measured on the Mindray™ with reagents from Gentian. TIMP‐2, KIM‐1, and NGAL levels were analyzed with the commercial sandwich ELISA kit, (DY1757, DY971, and DY1750B, R&D Systems, Minneapolis, MN). The total coefficient of variations for the ELISAS were approximately 6%.

Mean + 2 standard deviations (SD) of values from healthy volunteers where used as cut offs for U‐Cystatin C^12^ and TIMP‐2 and set accordingly to 0.414 mg/L for U‐Cystatin C and 7581 pg/mL for TIMP‐2. Urine albumin over 3 mg/mmol Creatinine was graded as microalbuminuria and over 30 mg/mmol Creatinine as severe albuminuria. Cut‐off values for U‐NGAL (100 ng/mL), and KIM‐1 (2.37 ng/mg Creatinine) were chosen from published values.^13^, ^14^

### 2.4 Statistical analysis

For statistical comparison with continuous‐dependent variables by group, Kruskal Wallis Rank Sum Test was used and for post hoc comparison pairwise Wilcoxon Rank Sum Tests with Bonferroni adjustment of *P*‐values. Categorical‐dependent variables by group were tested using Fisher's exact test or Chi square test when Fisher's exact test was not applicable. A probability of chance difference <.05 was considered significant. Missing values were excluded. Statistical calculations were made using R version 3.6.3. Continuous variables are presented as mean ± SD, frequencies are presented as absolute numbers/total participants (% of study population) if not stated otherwise.

## 3 RESULTS

### 3.1 Patient characteristics

**Figure 1:**
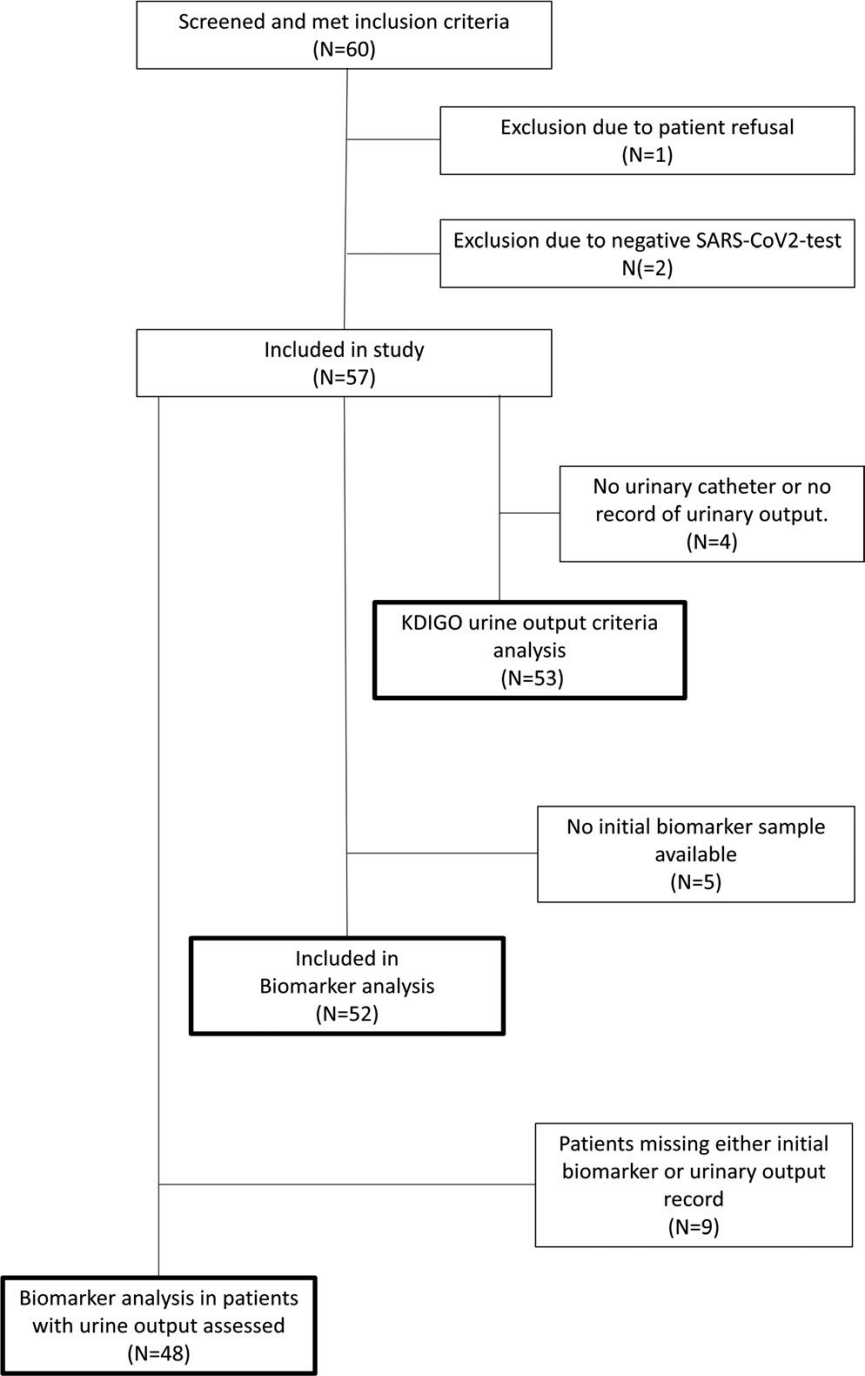
Flow chart detailing patient inclusion and analysis strata participation in a prospective observational study of acute kidney injury (AKI) incidence and biomarker profile in patients admitted with severe COVID‐19 in a tertiary ICU at a University Hospital in Sweden

Between March 13 and April 14, 2020, 60 patients were screened for eligibility of which 59 gave informed consent. Two patients were excluded from this analysis due to negative PCR for SARS‐COV‐2. The remaining 57 patients were admitted with confirmed severe COVID‐19. For two patients no records of hourly urine production were available, and two patients did not receive urinary catheters. These were classified according to Creatinine criteria only. For three patients, hourly urine output data up to the end of ICU day 1 was disregarded due to incompleteness. These all developed AKI and later fulfilled urine output criteria. A urine sample within the first day after inclusion was available in 52 patients (Figure 1). Baseline P‐Creatinine values older than 7 days prior to ICU‐admission was used for 25 patients.

The majority of patients were men (77%) with mean age of 59 (±14) years. The most common comorbidities were hypertension (54%), diabetes mellitus (28%) and chronic pulmonary disease (25%), 40% of the patients were treated with angiotensin‐converting enzyme inhibitors or angiotensin II receptor blocking drugs (ACEi/ARB) (Table 1). The mean baseline P‐Creatinine was 73 ± 20 µmol/L. Only 6 (11%) patients presented with a baseline P‐Creatinine above the reference interval (women 90 µmol/L, men 105 µmol/L). Symptoms of COVID‐19 started on average 11 ± 3 days before ICU admission. Invasive ventilation was started immediately after ICU admission in 18% of patients and in 68% some time during the ICU stay. Mean SAPS 3 score was 53 ± 9. Most patients were circulatory stable on arrival with a mean arterial pressure (MAP) of 93 ± 16 mm Hg. Although, four patients received vasopressor drugs at admission, no patients were hypotensive (MAP < 65 mm Hg). Patients that developed stage 3 AKI tended to be older, have higher body weight, and higher P‐Creatinine at admission.

**Table 1:** Patient background and clinical characteristics at ICU in 57 patients treated for severe COVID‐19 according to acute kidney injury (AKI) stage. Values presented as mean ± SD or percent of *N*

	No AKI	KDIGO 1	KDIGO 2	KDIGO 3
N	6	9	29	13
Age (y)	53 ± 22	63 ± 9	56 ± 15	67 ± 6
Sex (% female)	33	44	24	0
Body weight (kg)	78 ± 14	77 ± 16	89 ± 14	93 ± 27
BMI	25 ± 3	26 ± 8	30 ± 5	32 ± 7
Pulmonary disease (%)	17	33	31	8
Hypertension (%)	33	56	52	69
Heart failure (%)	0	11	3	0
Ischemic heart disease (%)	0	22	7	15
Malignant disease (%)	0	11	7	8
Diabetes (%)	0	44	28	31
ACEi/ARB treatment (%)	17	56	41	38
Baseline P‐Creatinine (µmol/L)	69 ± 14	69 ± 25	73 ± 21	80 ± 13
Elevated baseline P‐Creatinine (%)	0	11	21	8
At ICU admission				
SAPS 3 score	47 ± 5	58 ± 9	51 ± 10	55 ± 9
Glasgow coma scale 15 (%)	80	67	79	92
Heart rate	81 ± 9	96 ± 26	91 ± 13	86 ± 15
Mean arterial pressure	92 ± 14	99 ± 20	92 ± 17	92 ± 15
COVID day	11 ± 4	11 ± 4	11 ± 3	10 ± 3
P‐Creatinine at arrival (µmol/L)	79 ± 18	81 ± 34	83 ± 38	244 ± 443
Invasive ventilation (%)	0	33	17	8
Vasopressor treatment (%)	0	22	3	8
During ICU stay				
Highest P‐Creatinine (µmol/L)	89 ± 20	95 ± 28	116 ± 54	441 ± 410
Invasive ventilation (%)	17	67	69	92
30 day mortality (%)	17	33	17	54
Total volume of fluids at end of ICU day 1 (mL/kg)	72 ± 57	59 ± 22	54 ± 23	50 ± 15
Cumulative fluid balance at end of ICU day 1 (mL/kg)	27 ± 21	13 ± 19	19 ± 20	16 ± 19
Positive cumulative fluid balance at end of ICU day 1 (%)	83	89	76	69

Note

Conversion factors for units: plasma creatinine in μmol/L to mg/dL, ×0.0113.

Abbreviations: ACEi, angiotensin‐converting enzyme inhibitors; ARB, angiotensin II receptor blockers; BMI, body mass index; eGFR, estimated glomerular filtration rate; ICU, intensive care unit; SAPS 3, simplified acute physiological score III.

### 3.2 Fluid balance

Patients in general had a positive cumulative fluid balance at the end of the first whole day in ICU (ICU day 1) with a mean positive balance of 18 ± 19 mL/kg and a mean total volume of administered fluids of 57 ± 27 mL/kg. There were no significant differences between groups in absolute values or normalized to actual body weight, or the proportions of patients with negative fluid balance at end of ICU day 1. No significant differences in fluid balance were found in the subgroup of patients without AKI at admission at end of ICU day 1. There were also no significant differences in cumulative fluid balance at the time of KDIGO AKI stage criteria fulfilment between stages 1, 2, and 3.

### 3.3 Acute kidney injury incidence and outcome

Acute kidney injury according to the KDIGO definition occurred in most patients (n = 51/57, 89%) where 29/57 (51%) of patients had KDIGO stage 2, and 13/57 (23%) stage 3 AKI. Continuous renal replacement therapy (CRRT) was initiated in 9/57 (16%) patients at mean 152 ± 113 hours after admission. The indications for CRRT initiation were consequences of AKI in all patients. Mortality within 30 days of ICU admission was 28%. Mechanical ventilation during intensive care was associated with AKI (*P* = .01).

### 3.4 Acute kidney injury development according to different criteria and time

**Figure 2:**
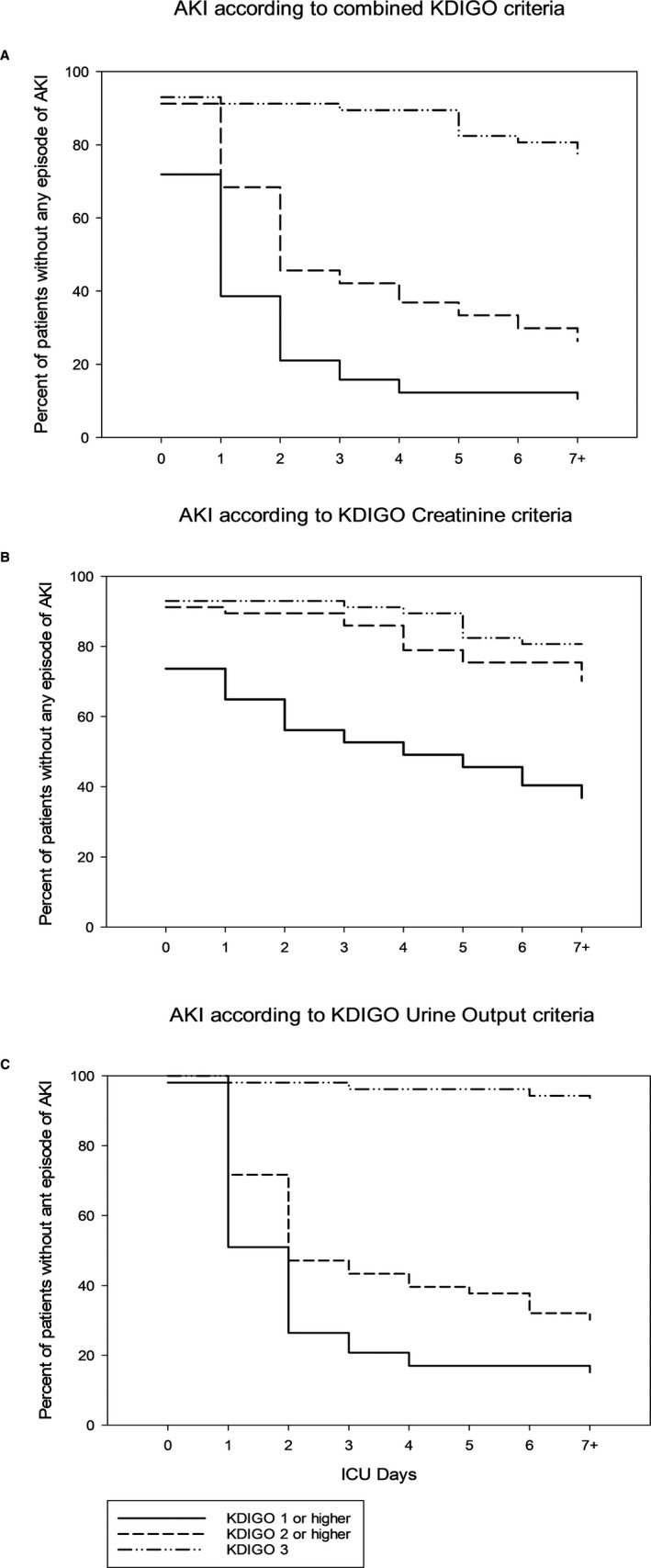
Cumulative incidence of acute kidney injury (AKI) as proportion of patients without AKI according different criteria by different stages in relation to ICU Days in 57 patients included in a prospective observational study of AKI incidence and biomarker profile in patients admitted with severe COVID‐19 in a tertiary ICU at a University Hospital in Sweden. Proportions are illustrated according to classification by (A) combined KDIGO criteria (B) KDIGO Creatinine criteria only and (C) KDIGO Urine Output criteria only (N = 53)

Acute kidney injury according to Creatinine criteria occurred in 36/57 (63%) of patients, whereas 44/57 (77%) of patients developed AKI according to urine output criteria (Table 2). Both Creatinine and urine volume criteria was fulfilled in 29/57 (51%) of patients. AKI severity by the two KDIGO criteria were, however, not well‐matched (Figure 2A‐C). A total of 27/57 (47%) patients had predominantly oliguric AKI and developed their highest AKI stage due to oliguria. Oliguria without fulfilment of Creatinine criteria occurred in 15/57 (26%) patients with AKI, and 12/57 (21%) of these developed KDIGO stage 2 AKI. Fulfilment of Creatinine criteria without oliguria occurred less frequently (n = 6, 9%).

**Table 2:** Matrix of relationship of number of patients by acute kidney injury (AKI) stages by two different criteria. Number of patients receiving continuous renal replacement therapy (CRRT) in brackets and patients without hourly urinary output records in parenthesis, in 57 patients included in a prospective observational study of AKI incidence and biomarker profile in patients admitted with severe COVID‐19 in a tertiary ICU at the University Hospital in Sweden. KDIGO_UO_ refers to Urine Output Criteria and KDIGO_Cr_ to Creatinine criteria

KDIGO_UO_ 3		1		3[2]
KDIGO_UO_ 2	12	11	5	4[4]
KDIGO_UO_ 1	3	3		2[2]
KDIGO_UO_ 0	6(3)	3(1)	1	3[1]
	KDIGO_Cr_ 0	KDIGO_Cr_ 1	KDIGO_Cr_ 2	KDIGO_Cr_ 3

Acute kidney injury according to the Creatinine criterion occurred in 15/57 (26%) patients before ICU admission (Figure 2B). Later development (on ICU day 3 or beyond) of AKI according to Creatinine criteria was common (12/36, 33%), especially for stage 2 and stage 3 AKI (12/18, 67%). Patients that developed oliguria severe enough to be classified as AKI tended to do so before the end of day 2 (37/44, 84%, Figure 2C). Finally, in patients without AKI at admission that later fulfilled the KDIGO AKI definition by both criteria, oliguria preceded Creatinine rise in 18 of 20 cases (90%) (*P* < .001).

### 3.5 Renal injury biomarkers

Albuminuria at ICU admission was common, and 38/52 patients (73%) had at least microalbuminuria, whereas 9/52 (17%) presented with severe albuminuria (Tables 3, 4, 5). There were no significant differences in U‐albumin between AKI Stages. U‐Cystatin C was elevated above cut‐off value in 39/52 (75%) of patients, in all groups more than 60% of patients had levels above cut off, whereas U‐NGAL was higher than 100 ng/mL in 13/52 (25%) patients. There were differences in Creatinine corrected U‐Cystatin C levels (*P* = .004) with the highest levels in stage 3 AKI (0.4 ± 0.4 mg/mmol) and lowest in stage 2 (0.1 ± 0.1 mg/mmol). In patients classified using urine output only with disregard of P‐Creatinine, stage 2 AKI was associated with lower levels of corrected U‐Cystatin C levels (0.1 ± 0.1 mg/mmol) compared to stage 1 (0.3 ± 0.2 mg/mmol) and no oliguria (0.3 ± 0.3 mg/mmol) (*P* = .01 and 0.01, respectively). Compared to the no oliguria group, stage 2 AKI using oliguria only, was also associated with lower proportions of NGAL above cut off (*P* = .03). KIM‐1 and TIMP‐2 were elevated above cut‐off value in 35/52 (67%) and 26/52 (50%) of patients, respectively, with no significant differences between groups in either absolute or corrected means or proportions above cut off.

**Table 3:** Levels and proportions of urinary biomarkers at ICU admission in different acute kidney injury (AKI) stages defined by combined KDIGO criteria. N = 52

	No AKI	KDIGO 1	KDIGO 2	KDIGO 3
N	6	9	27	10
U‐Albumin (mg/mmol)	15 ± 19	41 ± 66	10 ± 12	16 ± 13
Microalbuminuria (%)	50	78	70	90
Severe albuminuria (%)	33	33	7	20
U‐Cystatin‐C_Corr_ (mg/mmol)^††^	0.3 ± 0.3	0.2 ± 0.1**	0.1 ± 0.1**	0.4 ± 0.4
U‐Cystatin C (mg/L)	0.9 ± 0.7	1.2 ± 0.8	0.8 ± 0.6	1.6 ± 0.9
U‐Cystatin‐C > 0.414 mg/L (%)	83	89	63	90
U‐NGAL (ng/mL)	169 ± 221	95 ± 79	73 ± 140	239 ± 362
U‐NGAL_Corr_ (ng/mmol)	45 ± 82	27 ± 37	10 ± 24	60 ± 99
U‐NGAL > 100 ng/mL (%)	33	33	11	50
U‐KIM‐1 (ng/mL)	2.3 ± 3.3	3.2 ± 2.8	4.5 ± 3.8	3.5 ± 2.5
U‐KIM‐1_Corr_ (ng/mg)	2.5 ± 2.3	5.1 ± 3.8	5.3 ± 6.2	4.1 ± 2.6
U‐KIM‐1 > 2.37 ng/mg (%)	33	67	70	80
U‐TIMP2 (ng/mL)	6 ± 3	16 ± 29	9 ± 6	12 ± 9
U‐TIMP2_Corr_ (ng/mg)	10 ± 5	45 ± 99	10 ± 6	14 ± 9
U‐TIMP2 > 7.58 ng/mL (%)	20	20	64	56

Abbreviations: NGAL: Neutrophil Gelatinase‐Associated Lipocalin, KIM‐1, Kidney Injury Molecule 1, TIMP‐2 Plasma Tissue Inhibitor of MetalloProteinase 2.

Indicates difference between all groups (^††^ *P* < .01).

Indicates difference between groups (**P < .01).

**Table 4:** Levels and proportions of urinary biomarkers at ICU admission in different acute kidney injury (AKI) stages defined by KDIGO Creatinine criteria only. N = 52

	No AKICr	KDIGOCr 1	KDIGOCr 2	KDIGOCr 3
N	19	17	6	10
U‐Albumin (mg/mmol)	23 ± 47	12 ± 16	13 ± 19	16 ± 13
Microlbuminuria (%)	68	65	83	90
Severe albuminuria (%)	21	12	17	20
U‐Cystatin‐C_Corr_ (mg/mmol)	0.2 ± 0.2	0.1 ± 0.1	0.1 ± 0.1	0.4 ± 0.4
U‐Cystatin C (mg/L)	0.9 ± 0.7	0.8 ± 0.5	1 ± 0.7	1.6 ± 0.9
U‐Cystatin‐C > 0.414 mg/L (%)	74	71	67	90
U‐NGAL (ng/mL)	89 ± 134	60 ± 64	187 ± 279	239 ± 362
U‐NGAL_Corr_ (ng/mmol)	23 ± 52	11 ± 12	27 ± 49	60 ± 99
U‐NGAL > 100 (ng/mL) (%)	21	12	33	50
U‐KIM‐1 (ng/mL)	3.5 ± 3.6	4.2 ± 3.4	4.6 ± 4.1	3.5 ± 2.5
U‐KIM‐1_Corr_ (ng/mg)	3.7 ± 3.6	6.5 ± 7.2	3.9 ± 2.3	4.1 ± 2.6
U‐KIM‐1 > 2.37 ng/mg (%)	47	76	83	80
U‐TIMP2 (ng/mL)	8 ± 4	12 ± 21	14 ± 11	12 ± 9
U‐TIMP2_Corr_ (ng/mg)	9 ± 5	28 ± 73	14 ± 11	14 ± 9
U‐TIMP2 > 7.58 ng/mL (%)	47	41	67	60

Abbreviations: KIM‐1, Kidney Injury Molecule 1; NGAL, Neutrophil Gelatinase‐Associated Lipocalin; TIMP‐2 Plasma Tissue Inhibitor of MetalloProteinase 2.

There were no significant difference between groups.

**Table 5:** Levels and proportions of urinary biomarkers at ICU admission characteristics in different acute kidney injury (AKI) stages defined by KDIGO urine output criteria only. N = 48

	No AKIUO	KDIGOUO 1	KDIGOUO 2	KDIGOUO 3
N	8	8	29	3
U‐Albumin (mg/mmol)	19 ± 18	39 ± 70	9 ± 10	14 ± 17
Microalbuminuria (%)	75	75	69	100
Severe albuminuria (%)	25	25	7	33
U‐Cystatin‐C_Corr_ (mg/mmol)^†††^	0.3 ± 0.3**	0.3 ± 0.2**	0.1 ± 0.1**	0.6 ± 0.7
U‐Cystatin C (mg/L)	1.2 ± 0.8	1.5 ± 1	0.8 ± 0.5	1.7 ± 1
U‐Cystatin‐C > 0.414 mg/L (%)	88	88	62	100
U‐NGAL (ng/mL)	199 ± 264	211 ± 393	58 ± 77	172 ± 247
U‐NGAL_Corr_ (ng/mmol)^†^	53 ± 75	54 ± 84	6 ± 7	89 ± 146
U‐NGAL > 100 (ng/mL) (%)^†^	50*	38	10*	33
U‐KIM‐1 (ng/L)	2.1 ± 2.2	2.7 ± 3	4.8 ± 3.6	1.7 ± 1.7
U‐KIM‐1_Corr_ (ng/mg)	4 ± 3.7	4.5 ± 4	5.9 ± 4.9	2.8 ± 1
U‐KIM‐1 > 2.37 ng/mg (%)	63	50	76	67
U‐TIMP2 (ng/mL)	19 ± 31	7 ± 5	10 ± 7	6 ± 6
U‐TIMP2_Corr_ (ng/mg)	52 ± 104	15 ± 12	9 ± 4	12 ± 11
U‐TIMP2 > 7.58 ng/mL (%)	38	25	66	33

Abbreviations: KIM‐1, Kidney Injury Molecule 1; NGAL, Neutrophil Gelatinase‐Associated Lipocalin; TIMP‐2, Plasma Tissue Inhibitor of MetalloProteinase 2.

Indicates difference between all groups (^†^ *P* < .05, ^†††^ *P* < .0001).

Indicates difference between groups (*P < .05, **P < .01 between KDIGOuo 2 and KDIGOuo 0 + 1, respectively).

## 4 DISCUSSION

The main result of this study is that 89% of critically ill COVID‐19 patients admitted to intensive care developed AKI. A substantial group of patients exhibited predominantly oliguric early renal failure, where preserved GFR as estimated using plasma Creatinine was not uncommon. Furthermore, increased levels of urinary biomarkers of epithelial kidney injury, as well as increased urinary albumin excretion may indicate tissue damage at both the glomerular and tubular level. However, without clear relation between levels at admission and final AKI stage.

The finding that nine of 10 intensive care patients with COVID‐19 develop AKI is in agreement with a previous study^6^ but substantially higher than the proportion seen in general ICU populations without COVID‐19.^15^ In this material, the two main KDIGO criteria for AKI differed both regarding time trajectory and in the pattern of biomarkers. In patients with oliguria classified as AKI stage 2, isolated oliguria was common and lower levels of U‐Cystatin C and NGAL were found. Although little is known about the clinical relevance of oliguric renal failure in COVID‐19, several studies have investigated oliguria in the ICU and found that both duration and severity of oliguria is associated with increased risk of end‐stage renal disease and death^16^, ^17^, ^18^ but not in parity with the Creatinine criteria.^19^ This strengthen the notion that these pathways also benefit exploration individually.

Fluid overload is known to be associated with AKI in ARDS patients^20^ and too cautious hydration may cause oliguria as well. However, circulatory parameters at ICU admission in our study do not indicate that hypovolemia was common. Also fluid balance in general was positive after initial care, although not liberal. This is confounded by the fact that fluid balance prior to intensive care is not known and insensible losses aggravated by fever may be large. We have found instances of high renin values in combination with high levels of plasma sodium.^11^ Thus extra cellular fluid contraction aggravating AKI in COVID‐19 cannot be discarded. The optimal fluid management strategy to balance respiratory function and kidney function is not well‐studied and less so in COVID‐19.

A majority of patients with stage 3 AKI by Creatinine in this cohort received CRRT and biomarker levels in this group were high. It is common that AKI studies focus on AKI development within the first 48‐72 hours^21^, ^22^ after ICU admission, part due to the older Acute Kidney Injury Network (AKIN) criteria where an abrupt (within 48 hours) reduction in kidney function was part of the definition, but also to focus on AKI associated with the condition requiring intensive care rather than the effects of treatment. In this cohort, however, more severe AKI with reduced glomerular filtration rate tended to occur later. This is consistent with the long duration of stay seen in critically ill COVID‐19 patients, as well as the gradual progression of the illness during intensive care.^2^ This is in contrast to other reports where median time of RRT initiation from hospital admission was merely 2 hours, and 0 hours after AKI diagnosis was made.^6^ Reasons for this may include differences in hospital practices, population characteristics, and the prospective observational design of this study where AKI diagnosis was made independently from the treating clinician.

The present findings indicate that biomarkers at admission may not be easily applicable for AKI risk assessment in this cohort. Biomarkers generally tend to correlate well with AKI stage and each other in intensive care populations.^23^ One potential explanation to this discrepancy is the varying testing time point in relation to the start of the disease, where in AKI after cardio pulmonary bypass or ischemia/reperfusion injury the time and duration of the insult is generally known and can be tied to an early rise in biomarkers that may remain high for a time after the injury.^23^, ^24^ In the case of COVID‐19‐induced AKI the process is ongoing, which may indicate that biomarker development over time could be more informative, as has been shown in sepsis.^25^

We demonstrate a common occurrence of albuminuria in ICU‐treated COVID‐19 patients with frequent incidence of AKI. Albuminuria has recently been reported in relation to COVID‐19 patients but not previously considered in association with AKI.^26^ It is a risk factor for poor outcome in hospitalized patients,^3^ and is a known risk factor for development of chronic kidney disease after AKI.^27^ Thus, the present finding of albuminuria is worrisome and indicates a need for future studies investigating long‐term kidney disease progression after COVID‐19.

There are also limitations to this study. In the KDIGO definition of AKI, the problem of relating urine output to actual bodyweight was addressed but not resolved. It is probable that using lean or predicted bodyweight would reduce the incidence of AKI defined by urine output alone. Furthermore, true baseline Creatinine before contracting SARS‐COV‐2, but within a short time of admission were not generally available, as is generally the case in AKI studies. There is data missing regarding urine output both before ICU admission, and for some patients during intensive care. This may delay the time to urine output criteria fulfilment, and may underestimate the incidence of AKI. There is a relatively small number of patients and multiple comparisons are made, which combined with the low proportion of patients without AKI make more detailed analysis of the clinical usefulness of biomarkers hard to interpret. As a single center study local practices may influence outcome, however, ICU care at the department in question in general adheres to international standards and guidelines and is comparable to similar units in the region. Finally, urinary biomarkers were only analyzed after admission to ICU, whereas a theoretical window of clinical usefulness may occur before admission.

In conclusion, this study demonstrates a very high incidence of AKI in COVID‐19 patients that require intensive care. A large proportion of patients develop predominantly oliguric renal failure with initially less impact on plasma Creatinine. High‐stage AKI due to reduced GFR often occurred after several days of intensive care. Biomarker analysis exhibit elevated levels at admission, which may signal moderate tissue damage to both glomeruli and tubuli. The common occurrence of albuminuria highlights the need of studies evaluating long‐term renal outcome of COVID‐19 patients.

## CONFLICT OF INTEREST

The authors have no conflicts of interest.

## AUTHORS CONTRIBUTIONS

Luther T, Hultström M, Larsson A, Lipcsey M, and Frithiof R contributed to conception and design of the study. Luther T, Bülow Anderberg S, Frithiof R, Hultström M, and Lipcsey M collected patient data. Larsson A performed laboratory analyses. Luther T, Hultström M, Lipcsey M, and Frithiof R performed data analysis. The first draft of the manuscript was written by Luther T, Bülow Anderberg S, Frithiof R, Lipcsey M, and Hultström M. All authors commented on previous versions of the manuscript. All authors read and approved the final manuscript for publication.
